# Effects of a Low-Load Gluteal Warm-Up on Explosive Jump Performance

**DOI:** 10.1515/hukin-2015-0046

**Published:** 2015-07-10

**Authors:** Thomas Comyns, Ian Kenny, Gerard Scales

**Affiliations:** 1 Biomechanics Research Unit, University of Limerick, Limerick, Ireland.; 2 Irish Institute of Sport, Abbotstown, Dublin 15, Ireland.

**Keywords:** counter movement jump, squat jump, plyometrics, rest interval, rate of force development, speed strength

## Abstract

The purpose of this study was to investigate the effects of a low-load gluteal warm-up protocol on countermovement and squat jump performance. Research by Crow et al. (2012) found that a low-load gluteal warm-up could be effective in enhancing peak power output during a countermovement jump. Eleven subjects performed countermovement and squat jumps before and after the gluteal warm-up protocol. Both jumps were examined in separate testing sessions and performed 30 seconds, and 2, 4, 6 & 8 minutes post warm-up. Height jumped and peak ground reaction force were the dependent variables examined in both jumps, with 6 additional variables related to fast force production being examined in the squat jump only. All jumps were performed on a force platform (AMTI OR6-5). Repeated measures analysis of variance found a number of significant differences (p ≤ 0.05) between baseline and post warm-up scores. Height jumped decreased significantly in both jumps at all rest intervals excluding 8 minutes. Improvement was seen in 7 of the 8 recorded SJ variables at the 8 minute interval. Five of these improvements were deemed statistically significant, namely time to peak GRF (43.0%), and time to the maximum rate of force development (65.7%) significantly decreased, while starting strength (63.4%), change of force in first 100 ms of contraction (49.1%) and speed strength (43.6%) significantly increased. The results indicate that a gluteal warm-up can enhance force production in squat jumps performed after 8 minutes recovery. Future research in this area should include additional warm-up intervention groups for comparative reasons.

## Introduction

Improving an athlete’s explosiveness and power capabilities is a central objective of many training programs. The type of a warm-up undertaken prior to the performance of explosive movements, such as jumping or sprinting, can have an effect on the power output during these dynamic exercises. Research has been conducted to investigate the effect of warm-up protocols on subsequent explosive exercise performance with the aim of optimizing dynamic performance (Comyns et al., 2006; Crow et al., 2012; Gourgoulis et al., 2003; Saez Saez de Villareal, 2007). These protocols have incorporated heavy (Comyns et al., 2006), medium (Gourgoulis et al., 2003) and low (Crow et al., 2012) load exercises and often involved the performance of squat type exercises.

A large number of studies have investigated the acute effects of performing heavy resistance exercises as part of a warm-up protocol. Many of these have supported the efficacy of performing heavy loads (5 rep max, 5RM) of the back squat exercise in order to enhance subsequent jump performance (Comyns et al., 2006; Crewther et al., 2011; Gullick and Schmidtbleicher, 1996; Kilduff et al., 2007; Young et al., 1998). Despite these studies supporting the use of heavy squats, other studies have failed to achieve a performance-enhancing effect (Jensen and Ebben, 2003; Scott and Docherty, 2004; Witmer et al., 2010). These equivocal results may be due to a number of variables, such as rest interval post-squatting, that influence the benefits of the warm-up protocol (Docherty, 2004).

The gluteal muscles play a major role in running and jumping activities, and studies investigating lower limb muscular activity during running and jumping have found the gluteal muscles to be vigorously activated (Kyrolainen et al., 2005; Nagano et al., 2005; Palastanga et al., 2002). The hip extensor muscles have been proposed as the most important muscles for forward propulsion (Novacheck, 1998), while the gluteal muscles also play an important role in pelvic and trunk stability during movement (Weimann and Tidow, 1995). Subsequently Crow et al. (2012) investigated whether a warm-up consisting of low-load gluteal exercises could facilitate a warm-up effect on subsequent jump performance. This study by Crow et al. (2012) involved the completion of one set of ten of the following exercises: double leg bridge, quadruped lower extremity lift, quadruped hip abduction, side lying clams in 60° hip flexion, side lying hip abduction, prone single leg hip extension and stability ball wall squats. This gluteal protocol was compared to a whole-body vibration and control (no warm-up) protocol. Following completion of the warm-up the subjects were given 5 minutes rest before performing 5 consecutive countermovment jumps (CMJs) using an unweighted Smith machine bar. Peak power output (PPO) during the CMJs was recorded using a linear encoder; this was the only variable measured. PPO was found to be significantly higher following performance of the gluteal warm-up protocol when compared with a whole body vibration protocol (6.6% lower) and a control group (4.2% lower). The CMJ height was not recorded but the findings suggest that a low-load gluteal warm-up may be effective in enhancing explosive jump performance. The study did not investigate the optimum rest interval post warm-up or the effect of the warm-up routine on squat jump (SJ) performance.

In light of the existing research the present study sought to further investigate the effectiveness of the low-load gluteal protocol employed by Crow et al. (2012), by examining its effect on both CMJ and SJ performance. Almost all research investigating warm-up protocols and jump performance has been conducted using CMJs as the explosive jump activity. Minimal research has investigated the effect of a warm-up protocol on SJ performance. The SJ force-time trace can be analyzed to provide a number of measures of explosive force production, which are applicable to many elite sports where explosive power output of the lower limbs is one of the key determinants of performance (Izquierdo et al., 2002). The study also aimed to identify the optimal rest interval between completion of a gluteal warm-up protocol and peak jumping performance. Height jumped along with several measures of rapid force production is examined in the current study and act as the dependent variables.

## Material and Methods

### Participants

Eleven track and field athletes (six males and five females) formed the subject base for this study (age: 20.9 ± 2.6 years; body height: 175.6 ± 9.8 cm; body mass: 68.4 ± 7.0 kg). Seven of the athletes specialized in sprint events (100 m, 200 m or 400 m), one in the high jump, one long jump, one pole vault and one in the heptathlon. All subjects had previously competed at the national level in their event, with one subject having also competed internationally. The athletes were proficient in the technique of the CMJ and SJ, with both exercises having formed a part of their regular training for a minimum of 2 years. The subjects were injury-free and were participating in pre-season training at the time of the study. Approval from the University of Limerick Ethics Committee was received prior to recruitment. Subjects were informed of the experimental risks and signed an informed consent form and a Physical Activity Readiness Questionnaire before the investigation.

### Procedures

The experiment involved three testing sessions at equal intervals over a three-week period. For reliability reasons, and to control for circadian variation, each subject completed all three testing sessions on the same day of the week and at the same time (Atkinson and Reilly, 1996). Each testing session began with the same pre-intervention warm-up procedure consisting of four minutes of low-intensity aerobic exercises that involved jogging and skipping, followed by dynamic stretching of each of the major muscle groups of the lower body. This pre-intervention warm-up procedure was the same for each subject and for each testing day.

Testing session one was a familiarization session in which the subjects completed the aforementioned warm-up, followed by a number of CMJ and SJ trials until the experimenter was satisfied with their technique. For the CMJ the subjects were instructed to start from a straight leg position with hands placed on the hips, squat down to a self-selected depth before exploding upwards in an attempt to gain maximum height. They were instructed that this should be a smooth action with no pause in the crouch position. For the squat jump subjects were instructed to start by squatting to a 90° angle at the knee and hold that position until given the cue to jump by the experimenter, at which point they were to explode forcefully upwards and jump for maximum height. No further dip or counter movement was permitted as only an upward motion constitutes a correct SJ. In both jumping exercises subjects were instructed to keep both legs extended while in the air and land back on the force platform before bending their knees. The force-time trace for each SJ was examined to ensure that no dip occurred at the beginning of the contraction as this would indicate a counter movement. To conclude the familiarization session the subjects were asked to perform the low-load gluteal warm-up protocol and instructed on the correct technique for each of the seven exercises. The present study replicated the low-load gluteal warm-up protocol employed by Crow et al. (2012). The protocol consisted of seven exercises, with one set of 10 repetitions performed for each one. The exercises were a double leg bridge, quadruped lower extremity lift, quadruped hip abduction, side lying clams in 60° hip flexion, side lying hip abduction, prone single leg hip extension and stability ball wall squats (Table 1). Each movement was held for one second before returning to the starting position and progressing to the next repetition. Fifteen seconds rest was given after each exercise. The entire intervention warm-up protocol took approximately seven minutes to complete.

**Figure 1:**
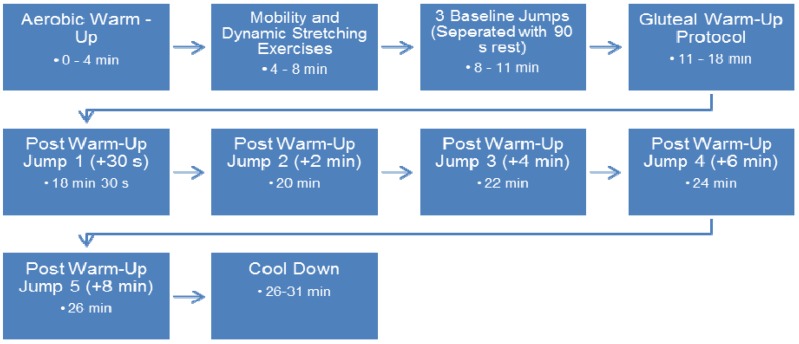
Timeline of Testing Sessions 2 and 3.

In the second testing session the subjects completed the general warm-up followed by recording of three baseline CMJs. The baseline jumps were separated with ninety seconds rest. The subjects then performed the gluteal warm-up followed by a further five CMJs, one at each of the pre-determined rest intervals (30 s, 2 min, 4 min, 6 min and 8 min). Subjects then completed a cool-down consisting of light jogging and static stretching of each of the major lower body muscle groups. Testing session three followed the same procedure as session two except the CMJs were replaced by SJs. A timeline of testing sessions 2 and 3 is illustrated in Figure 1.

### Instrumentation

All jumps were performed on an AMTI force platform (AMTI OR6-5), mounted flush with the surrounding laboratory floor. Data were collected with the system sampling at 1000 Hz. Force-time traces were real-time displayed and saved with the use of computer software (AMTI NetForce 2.4.0, Watertown, MA) for further analysis. Reliability of this particular force platform had previously been established (Comyns et al., 2006). A pilot study was conducted to assess reliability of the testing procedures. For the pilot study participants were asked to complete eight consecutive CMJs or SJs (three baseline, followed by five minutes rest, followed by one at each of the testing rest intervals). The intraclass correlation coefficients (ICC) of these jumps were then analyzed for all trials for both jumps to confirm reliability and that there would be no potentiation or fatigue effect on each jump from the performance of the preceding jumps during the experiment. The reported ICCs were as follows: CMJ: ICC = 0.951; SJ: ICC = 0.961. The coefficient of variation for all trials for the pilot CMJ and SJ were 3.3% and 3.8%, respectively. These results would indicate that any changes in jump performance were due to the gluteal warm-up intervention.

### Measures

The dependent variables for the CMJ were height jumped and peak ground reaction force (GRF). Peak GRF was calculated from the force-time CMJ traces. Jump height was derived from the flight time score, obtained via inspection of the force-time CMJ traces. The SJ force platform data were used to calculate jump height, peak GRF, time to peak GRF, max RFD, time to max RFD, starting strength, change in force in first 100 ms and speed strength index. The details of how each variable was derived are detailed in Table 2. Prior to the calculation of these variables the start of concentric contraction for the SJ was established. It was defined as the point at which the force readings were 10 N greater than the average of the force readings when the subject was static in the SJ starting position (Harrison and Bourke, 2009). These SJ variables had previously been utilized in research. Harrison and Bourke (2009) investigated the effect of resistance type training on SJ performance and employed the variables max RFD, time to max RFD and starting strength.

### Statistical Analyses

All statistical analyses were conducted using IBM SPSS Statistics (Release 20.0.0). The differences between the average of the three baseline scores for each dependent variable and the scores after each recovery interval were evaluated individually using a repeated-measure GLM ANOVA. The ANOVA had 1 within-subjects factor, namely Condition, with 2 levels (baseline and one of 30 s, 2, 4, 6 or 8 min). This analysis was performed on all recorded variables in both the CMJs and SJs.

Effect sizes using Cohen’s *d* (1988) were also obtained for each variable found to be significantly different from baseline using the following formula:

$$d=\frac{M_{1}-M_{2}}{\sigma_{\mathrm{pooled}}}$$

(M_1_ = mean of group 1; M_2_ = mean of group 2; *σ*_pooled_ = pooled standard deviation)

The pooled standard deviation is found as the root mean square of the two standard deviations:

$$\sigma_{\mathrm{pooled}}=\sqrt{\frac{{\sigma_{1}}^{2}+{\sigma_{2}}^{2}}{2}}$$

(*σ*_1_ = standard deviation of group 1; *σ*_2_ = standard deviation of group 2)

Effect sizes were interpreted using the scale suggested by Cohen (1988). According to Cohen (1988), an effect size less than 0.2 is trivial, between 0.2 and 0.5 small, between 0.5 and 0.8 medium, between 0.8 and 1.3 large and an effect size greater than 1.3 is very large.

## Results

**Figure 2:**
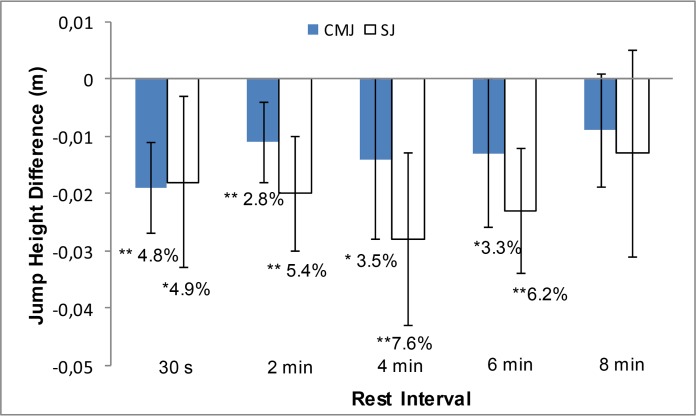
Mean ± 95% CI Height Jumped difference between the baseline CMJs and SJs and the CMJs and SJs at each different rest interval. ^***^p<0.001; ^**^p<0.01; ^*^p<0.05.

**Figure 3:**
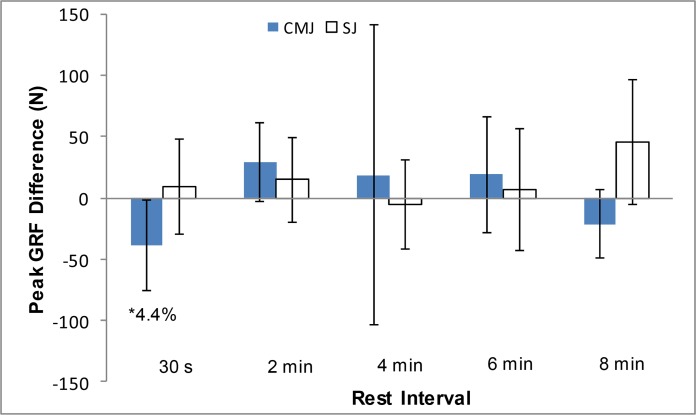
Mean ± 95% CI Peak Ground Reaction Force difference between the baseline CMJs and SJs and the CMJs and SJs at each different rest interval. ^*^p<0.05.

The results for FT and peak GRF are presented in Figures 2 and 3. The mean baseline scores for FT and GRF were subtracted from their corresponding post intervention scores at each rest interval. Thus in Figures 2 and 3 the x-axis represents the baseline scores. Figure 2 illustrates the results for height jumped for the CMJ and SJ. The GLM ANOVA CMJ results showed a significant reduction in performance after 30 s (p<0.0001; *d*=0.305, small), 2 min (p=0.006; *d*=0.177, trivial), 4 min (p=0.05; *d*=0.218, small) and 6 min (p=0.049; *d*=0.227, small) rest. Similar to the CMJ, the analysis of height jumped for the SJ showed a significant decrease in performance after 30 s (p=0.023; *d*=0.367, small), 2 min (p=0.001; *d*=0.326, small), 4 min (p=0.002; *d*=0.436, small) and 6 min (p=0.001; *d*=0.379, small) rest, as shown in Figure 2.

The peak GRF results for the CMJ and the SJ are presented in Figure 3. Statistical analysis showed a significant decrease in CMJ peak GRF at the 30 s rest interval (p=0.043; *d*=0.238, small). The mean CMJ peak GRF increased at the 2, 4 and 6 min interval, before decreasing again after 8 min, however the GLM ANOVA did not report these differences as significant. Mean SJ peak GRF scores increased from baseline at all rest intervals, excluding 4 min, however the GLM ANOVA did not report these differences to be significant (p>0.05).

The results pertaining to the SJ fast force production variables are presented in Table 3. The baseline data and the data referring to the difference between the baseline scores and the scores at each recovery interval are provided in this table. Any differences that were statistical significant are highlighted. Statistical analysis showed a significant increase in max RFD at the 2 min rest interval (p=0.006; *d*=0.498, small). Max RFD also increased at all other rest intervals, however these increases were not deemed significant (p>0.05). Time to peak GRF and time to peak RFD showed an improvement in both variables at the 8 min rest interval, with a significant 43% reduction in the time taken to reach peak GRF (p=0.031; *d*=1.007, large) and 65.7% reduction in the time to max RFD (p=0.042; d=0.998, large). The statistical analysis also showed a significant 63.4% increase in starting strength for the 8 min rest interval (p=0.002; Journal of Human Kinetics - volume 46/2015 *d*=0.839, large) and a significant increase of 49.1% in the change in force in the first 100 ms at this recovery interval (p=0.013; *d*=0.802, large). Finally, statistical analysis of the speed strength results also showed a significant 43.6% increase after 8 min rest (p=0.004; *d*=0.955, large).

## Discussion

The results of this investigation provide insight into the effects of a low-load gluteal warm-up on explosive jump performance. The height jumped results for both the CMJ and SJ showed a significant reduction compared to baseline scores at the 30 s, 2 min, 4 min and 6 min rest intervals, suggesting that the warm-up protocol elicited fatigue rather than performance enhancement in the athletes. In order to understand the true effects of the low-load gluteal warm-up routine on explosive jump performance, a number of other important variables were also examined. While height jumped is the outcome measure of jump performance, other variables recorded in this study provide insight into the jumping process and the generation of impulse. Significant improvements were seen in many of the explosive SJ variables. Seven of the eight variables recorded during SJ performance showed improvement at the 8 min rest interval, with analysis finding five of these improvements to be statistically significant. These improvements in explosive ability may be of great interest to those participating in sports where rapid force production is required.

As shown in the results section height jumped in both the CMJ and SJ decreased significantly after 30 s, 2 min, 4 min and 6 min of rest. A decrease in height jumped was also evident after 8 min, however this decrease was of a small magnitude (CMJ: 2.3% lower and SJ: 3.5% lower) and was not deemed significant by statistical analysis. The height jumped variable in both jumping exercises demonstrated a trend towards recovery to baseline values as the rest interval increased. Previous research has found squat warm-up protocols to have a detrimental effect on height jumped in CMJs performed immediately after the resistance exercise (Comyns et al., 2006; Crewther et al., 2011; Jensen and Ebben, 2003; Kilduff et al., 2007; Lowery et al., 2012). However, unlike the present study in which fatigue remained evident up to 6 min after completion of the warm-up, in previous studies CMJ performance at subsequent rest intervals was found to either show a non-significant decrease, return to baseline scores or exhibit a potentiation effect. This result is surprising, as an advantage of a low-load gluteal warm-up, as claimed by Crow et al. (2012), is that it is less fatiguing than the 3RM or 5RM protocols utilized in the aforementioned studies. In light of this result, it may be proposed that consecutive exercises isolating a single muscle group may be more fatiguing than a compound exercise targeting a greater number of muscles, albeit with a heavier load. Further research is required to investigate the effects of the gluteal warm-up beyond the 8 min examined in the present study. It would be of interest to observe whether, after more than 8 min rest, fatigue subsided and height jumped returned to baseline or improved.

The peak GRF scores in both CMJs and SJs in the present study provided limited significant results. Similar to previous research (Comyns et al., 2006; Jensen and Ebben, 2003) the peak GRF variable for the CMJ was significantly decreased 30 s following completion of the warm-up protocol. Peak GRF values increased, although not significantly, after 2, 4 and 6 min rest, before showing a reduction from baseline at the 8 min interval. For the SJ, peak GRF values increased for all trials excluding the 4 min rest interval, with statistical analysis revealing no significance in any of these changes.Maximum force is rarely reached in sprinting or jumping events and so peak GRF values are of less importance to those participating in explosive sporting activities than the various different measures of explosiveness such as max RFD, starting strength and time to peak GRF.

Had the present study focused solely on height jumped as a measure of jump performance the efficacy of gluteal activation may have been rejected. However, by investigating the process of the jump, considerable evidence emerged among the explosive and RFD variables supporting its usefulness as a pre-training or competition warm-up routine for activities requiring rapid force production. The results indicate that a specific gluteal warm-up can also be effective in improving force production in the early phase of muscle contraction in SJs, with time to peak GRF (43.0%), time to max RFD (65.7%), starting strength (63.4%), change of force in first 100 ms of contraction (49.1%) and speed strength (43.6%) all showing significant positive improvement at the 8 min rest interval. Max RFD was improved at every post warm-up rest interval, however statistical analysis deemed only the improvement after 2 min rest as significant.

RFD parameters have important functional significance in fast and forceful muscle contraction (Aagard et al., 2002). It takes ≥300 ms for human knee extensor muscles to reach maximum force (Thorstensson et al., 1976). In contrast, rapid movements involved in sprinting or jumping activities involve short contraction times. Tidow (1990) reported typical contraction times to be 80–100 ms for sprinting and 120–190 ms for jumping events (high jump, long jump and pole vault). Therefore, during fast limb movements maximal force is rarely, if ever, reached. This means any increase in RFD is vital in allowing athletes to reach a higher level of muscle force in the early phase of muscle contraction and thus increasing the impulse (Aagard et al., 2002). An impulse is the product of force and time and is represented as the area underneath the force-time curve (Hall, 2012). One objective of explosive training programs is to improve RFD, moving the force-time curve up and to the left (making it sharper and steeper in appearance), and in turn generating a greater impulse. A greater impulse during contraction allows for more explosive power output, one of the key determinants of performance in elite sports involving jumping and sprinting activities (Izquierdo et al., 2005). The improvements recorded in RFD and other explosive variables in this investigation suggest that performing gluteal activation exercises prior to competition could prove useful in enhancing performance in sporting events requiring impulse generation through explosive force production. Of interest for future research would be to compare the gluteal warm-up investigated in this study to both a control group and a dynamic warm-up group. A limitation in the present study is the lack of comparison to other warm-up interventions. Future research should address this by the inclusion of additional warm-up intervention groups within the experimental design.

## Conclusion

The results of the current study suggest that a low-load gluteal warm-up is effective in enhancing fast force production variables related to SJ and CMJ performance. The results are applicable to those participating in sports where explosive force production is necessary to optimize performance, for example sprinting and jumping. No improvement, however, is evident for CMJ or SJ height jumped post the gluteal warm-up indicating that such a protocol may be inappropriate for sports where maximum height is the key contributor to performance.

The warm-up employed in this study required little equipment and thus can be easily incorporated into pre-training and competition routines. Large and statistically significant improvements in performance were recorded at the 8 min interval across a number of measures of fast force production. Therefore, in order to enhance dynamic performance in rapid movement activities a gluteal warm-up could be performed 8 min prior to commencement of the event. For comparative reasons, future research should include additional warm-up intervention groups within the research design.

## Figures and Tables

**Table 1:** Gluteal Warm-Up Protocol Including EMG Muscle Activation Levels (adapted from Crow et al., 2012).

***Exercise***	***As described by***	***Gluteus Maximus (%MVIC)***	***Gluteus Medius (%MVIC)***
Double Leg Bridge	Ekstrom et al. (2007)	25 ± 14	28 ± 17
Quadruped Lower Extremity Lift	Ekstrom et al. (2007)	42 ± 17	56 ± 22
Quadruped Hip Abduction	American Council on Exercise	N/A	N/A
Side Lying Clam (60° flexion)	Di Stefano et al. (2009)	39 ± 34	38 ± 29
Side Lying Hip Abduction	Ekstrom et al. (2007)	21 ± 16	39 ± 17
Prone Single Leg Hip Extension	Lewis and Sahrmann (2009)	22 ± 10	N/A
Stability Ball Squat	American Council on Exercise	N/A	N/A

MVIC = maximum voluntary isometric contraction; N/A = EMG data not available

**Table 2:** Unit of measurement and method of calculation of dependent variables

***Dependent Variable***	***Unit***	***Method of Calculation***
Height Jumped (CMJ and SJ)	Metres (m)	(9.81 × Flight time^2^) / 8
Peak Ground Reaction Force (SMJ and SJ)	Newton (N)	Maximum force value from start of contraction to take-off point
Time to Peak Ground Reaction Force (SJ only)	Milliseconds (ms)	Time difference from start of contraction to peak GRF
Maximum Rate of Force Development (SJ only)	Newton per second (N·s^−1^)	Greatest rise in force over 5 ms between start of contraction and peak GRF (Tidow, 1990)
Time to Maximum Rate of Force Development (SJ only)	Milliseconds (ms)	Time between start of contraction and the beginning of maximum RFD (Tidow, 1990)
Starting Strength (SJ only)	Newton (N)	Difference between the force at the start of contraction & 30 ms later (Tidow, 1990)
Change of Force in First 100ms (SJ only)	Newton (N)	Difference between the force at the start of contraction & 100 ms later (Tidow, 1990)
Speed Strength (SJ only)	Newton per second (N·s^−1^)	Peak GRF divided by time to peak GRF (Tidow, 1990)

**Table 3:** Squat jump performance indicators comparing baseline with data 30 s, 2 min, 4 min, 6 min and 8 min post baseline measurement. Significant absolute percentage change from baseline is noted where appropriate.

			**Difference From Baseline**				
		**Baseline**	**30 s**	**2 min**	**4 min**	**6 min**	**8 min**
**Max RFD (N·s^−1^)**	**Mean**	9275.07	962.64	1531.77	280.19	1062.23	1943.73
	**Sig.**		0.059	0.006^*^	0.589	0.255	0.054
	**95% Level**		1011.30	955.73	1131.98	1978.11	1982.61

**Time To Peak GRF (ms)**	**Mean**	309.05	−99.86	−99.32	3.35	75.41	132.97
	**Sig.**		0.128	0.078	0.966	0.495	0.031^**^
	**95% Level**		136.73	113.20	171.80	240.08	117.35

**Time to Max RFD (ms)**	**Mean**	208.30	105.38	−102.20	17.90	66.90	−136.80
	**Sig.**		0.157	0.114	0.821	0.544	0.042^***^
	**95% Level**		157.33	132.05	174.16	240.02	130.36

**Starting Strength (N)**	**Mean**	116.27	−5.10	3.20	−16.22	−19.11	73.66
	**Sig.**		0.745	0.91	0.274	0.514	0.002^a^
	**95% Level**		35.60	62.05	31.51	63.66	38.06

**Change of Force in first 100ms (N)**	**Mean**	508.42	24.14	12.81	−8.27	−87.82	249.85
	**Sig.**		0.629	0.884	0.845	0.469	0.013^b^
	**95% Level**		112.88	193.45	93.02	262.76	182.32

**Speed Strength (N·s^−1^)**	**Mean**	7488.31	567.70	754.68	265.82	−491.71	3263.65
	**Sig.**		0.24	0.366	0.603	0.709	0.004^c^
	**95% Level**		1045.14	1793.02	1117.14	2882.80	1953.93

* p<0.01, 16.5%

** p<0.05, 43.0%

*** p<0.05, 65.7%

a p<0.01, 63.4%

b p<0.05, 49.1%

c p<0.01, 43.6%
